# Molecular Predictors of Long-Term Survival in Glioblastoma Multiforme Patients

**DOI:** 10.1371/journal.pone.0154313

**Published:** 2016-04-28

**Authors:** Jie Lu, Matthew C. Cowperthwaite, Mark G. Burnett, Max Shpak

**Affiliations:** 1 NeuroTexas Institute Research Foundation, St. David’s Healthcare, Austin, Texas, United States of America; 2 Center for Systems and Synthetic Biology, University of Texas, Austin, Texas, United States of America; 3 Fresh Pond Research Institute, Cambridge, Massachusetts, United States of America; Baylor College of Medicine, UNITED STATES

## Abstract

Glioblastoma multiforme (GBM) is the most common and aggressive adult primary brain cancer, with <10% of patients surviving for more than 3 years. Demographic and clinical factors (e.g. age) and individual molecular biomarkers have been associated with prolonged survival in GBM patients. However, comprehensive systems-level analyses of molecular profiles associated with long-term survival (LTS) in GBM patients are still lacking. We present an integrative study of molecular data and clinical variables in these long-term survivors (LTSs, patients surviving >3 years) to identify biomarkers associated with prolonged survival, and to assess the possible similarity of molecular characteristics between LGG and LTS GBM. We analyzed the relationship between multivariable molecular data and LTS in GBM patients from the Cancer Genome Atlas (TCGA), including germline and somatic point mutation, gene expression, DNA methylation, copy number variation (CNV) and microRNA (miRNA) expression using logistic regression models. The molecular relationship between GBM LTS and LGG tumors was examined through cluster analysis. We identified 13, 94, 43, 29, and 1 significant predictors of LTS using Lasso logistic regression from the somatic point mutation, gene expression, DNA methylation, CNV, and miRNA expression data sets, respectively. Individually, DNA methylation provided the best prediction performance (AUC = 0.84). Combining multiple classes of molecular data into joint regression models did not improve prediction accuracy, but did identify additional genes that were not significantly predictive in individual models. PCA and clustering analyses showed that GBM LTS typically had gene expression profiles similar to non-LTS GBM. Furthermore, cluster analysis did not identify a close affinity between LTS GBM and LGG, nor did we find a significant association between LTS and secondary GBM. The absence of unique LTS profiles and the lack of similarity between LTS GBM and LGG, indicates that there are multiple genetic and epigenetic pathways to LTS in GBM patients.

## Introduction

Glioblastoma multiforme (GBM) is the most frequent malignant form of primary brain cancer in adults. The median survival time for GBM patients is approximately 14 months with intensive multimodal therapy that includes surgical resection, chemotherapy, and radiotherapy [1] after initial diagnosis. GBM can develop both *de novo* or via progression from primary low grade glioma (LGG). A small proportion of patients survive for exceptionally long periods of time; for example, fewer than 10% of GBM patients survive more than 3 years [2–4]. Therefore, studying the clinical and molecular characteristics of these rare instances of long-term survival (LTS) among GBM patients may provide insights into both the molecular basis of GBM progression and identify potential new prognostic biomarkers.

Several patient characteristics like age, performance status and tumor localization have been identified as predictors of survival time [4–7]. However, many studies have often attributed the causes of LTS in GBM to erroneous histopathological diagnosis (i.e. misidentification of low-grade gliomas as GBM) or as statistical anomalies [8–10]. With advances in microarray and sequencing technologies, associations of molecular markers such as mutations, gene expression levels, DNA methylation states, and microRNAs with LTS tumors have been reported [2, 3, 11–21]. Using these techniques, *MGMT* hypermethylation and mutations in isocitrate dehydrogenase (*IDH1*) have been the most frequently identified genomic marker of improved patient response to chemotherapy and therefore longer patient survival [12, 22, 23]. Unfortunately, many of these studies have only independently considered a single class of molecular marker, so that integrative studies of multiple types of molecular marks specifically associated with LTS are still lacking. In [21], the transcriptional profiles of 7 LTS patients were compared to non-LTS, their study found no association between LTS and transcriptional subtype. The analysis in [21] was qualitative; there have been no model-based analyses integrating different classes of genomic data to systematically determine whether LTS cases simply represent extreme outliers of a distribution defined by what is effectively a single pathology, or, alternatively, if they represent a biologically distinct class of GBMs with unique genomic, epigenetic, and phenotypic characteristics. Similarly, other analyses (e.g. [24]) have identified correlations between genomic alterations and GBM patient survival times, the analysis and markers were not specific to LTS patients.

If LTS cases do have unique molecular characteristics among GBMs, there is also the potential for similarity between LTS GBM and LGG at the molecular level. There are two reasons to seriously investigate the hypothesis that LTS GBMs share molecular profiles with LGG. First, it is often suggested that many LTS GBMs are misdiagnosed instances of LGG. Second, the best-known genomic predictors of improved responses to temozolomide (TMZ) chemotherapy are mutations in *IDH1* and methylation of the *MGMT* promoter, which are themselves frequently associated with secondary GBM (those that have progressed from LGG, as opposed to *de novo* GBM [25]). These and similar observations suggest that secondary GBMs may retain additional genomic traits and have clinical features that are more typical of LGG. We will determine whether this is indeed the case, and whether these secondary GBMs are also significantly characterized by LTS.

In this study, we leverage 6 types of molecular data from The Cancer Genome Atlas (TCGA) GBM samples, including germline and somatic point mutation, gene expression, DNA methylation, copy number variation (CNV) and microRNA (miRNA) expression data. We use machine learning to identify molecular markers characteristic of LTS in GBM, and construct integrative models that incorporate multiple sets of molecular profiles that are jointly predictive of LTS when combined with clinical and demographic data. We also explore whether LTS cases of GBM have molecular characteristics typical of LGG by comparing the similarity of LTS tumor genetic profiles to LGG. The results of our analyses have important implications for our understanding of the molecular pathology of GBM, as well as providing insight into the design of novel prognostic and therapeutics indicators.

## Material and Methods

### Classification of LTS Phenotypes

Clinical and demographic data describing patient age at initial diagnosis, gender, ethnicity/race, treatment history, vital status and follow-up/survival times were collected from TCGA [26] (Table 1). We used Kaplan-Meier survival analysis to build survival curves and identify LTS patients. In keeping with criteria for LTS used in the clinical literature (e.g. [2–4]), we used a three-year threshold survival time, regardless of vital status, in order to classify patients as LTS. Actual survival times were not used in the analysis, only the binary LTS/non-LTS classification. Only patients with documented survival times of less than 3 years were considered to be non-LTS patients in our study; patients with uncertain status beyond the 3 year point were excluded. For comparison, a more stringent cutoff of 1615 days (4.5 years, defining the upper 5% survival time in TCGA’s GBM data set) was also applied as an alternative criterion to classify patients as LTS and non-LTS. We also considered GBM cases with a prior history of LGG as a separate class of data, i.e. these secondary GBMs represent a set of patients who, in contrast to most GBM cases, have likely previously received chemotherapy and/or radiation therapy for the earlier cancer. Both treatment types have the potential to induce distinct genetic and epigenetic profiles.

**Table 1:** Summary of clinical and demographical information of the TCGA patient cohort used for this study.

Total number of patients	591
Clinical outcomes	
Overall survival	0–10.6 years
Median survival	0.9 year
Event(Alive/Dead)	146/443
Classifications	
LTS (survival > 3 years)	44
nonLTS(survival < 3 years and dead)	411
Censored (survival < 3 years and alive)	136
Clinical Covariates	
Age at initial diagnosis	10–89 (median 59)
Race (white/Asian/Black)	503/13/50
Gender (Female/Male)	228/363
History of LGG diagnosis (Yes/No)	15/576

### Genomic data processing

All genomic/molecular data, including exome sequences, germline mutations, probes for microarray gene expression, DNA methylation, CNV and miRNA expression were retrieved from TCGA for both GBM and LGG patients. The platforms and levels of data are summarized in Table 2. Somatic point mutations were called from the whole exome bam files using the pipeline described in [27], where SomaticSniper [28] was used to call mutations, the output was filtered for read quality with a custom Python script. Somatic point mutations were classified as missense, nonsense, silent, etc. using snpEff [29]. Non-silent somatic mutations that were identified in more than one tumor were retained as candidate predictors of survival time for subsequent inclusion in our models. Additionally, germline single nucleotide polymorphisms (SNPs) were identified from the Level II TCGA SNP data, these were processed to exclude low quality genotype calls and rare alleles, using a pipeline described in [30].

**Table 2:** Summary of the 6 types of molecular data and their platforms used for this study.

	Platform	Number of patients	LTS/non-LTS	Total features	ULR features	LLR features
Germline Mutation	Affimetrix Genome Wide SNP6 array	346	30/316	532954	0	0
Somatic Mutation	Illumina Genome Analyzer DNA Sequencing	187	18/169	1419	10	13
Gene expression	Affymetrix Human Genome U133 Plus 2.0 Array	415	39/376	22277	38	94
DNA methylation	Illumina Infinium Human DNA Methylation 27/450	283	22/261	23233	38	43
miRNA	Agilent 8X15 k human miRNA-specific microarray	437	43/394	534	1	1
CNV	Agilent Human Genome CGH Microarray 244A	417	43/374	23169	11	29

Level 1 gene expression data were collected for GBM, LGG, and normal tissue samples from TCGA. The expression data was RMA adjusted [31] and transformed to a base-2 logarithmic scale. Level 3 DNA methylation data from two platforms (Illumina methylation arrays 27 and 450) were combined by intersecting the probe sets, excluding 10.1% of samples with more than 5% missing values. Missing values in the remaining probes were imputed using the median value across samples. The level 3 miRNA expression data was used without any further processing. For the Level 3 CNV data, a weighted average CNV score was computed if a gene spanned multiple segments of a CNV probe, with score weights proportional to the fraction of the gene spanned by each probe. Unless otherwise indicated, the data processing and all analyses were implemented in Python 2.7.5 and R 3.0.3.

### Regression Analysis

**Fig 1:**
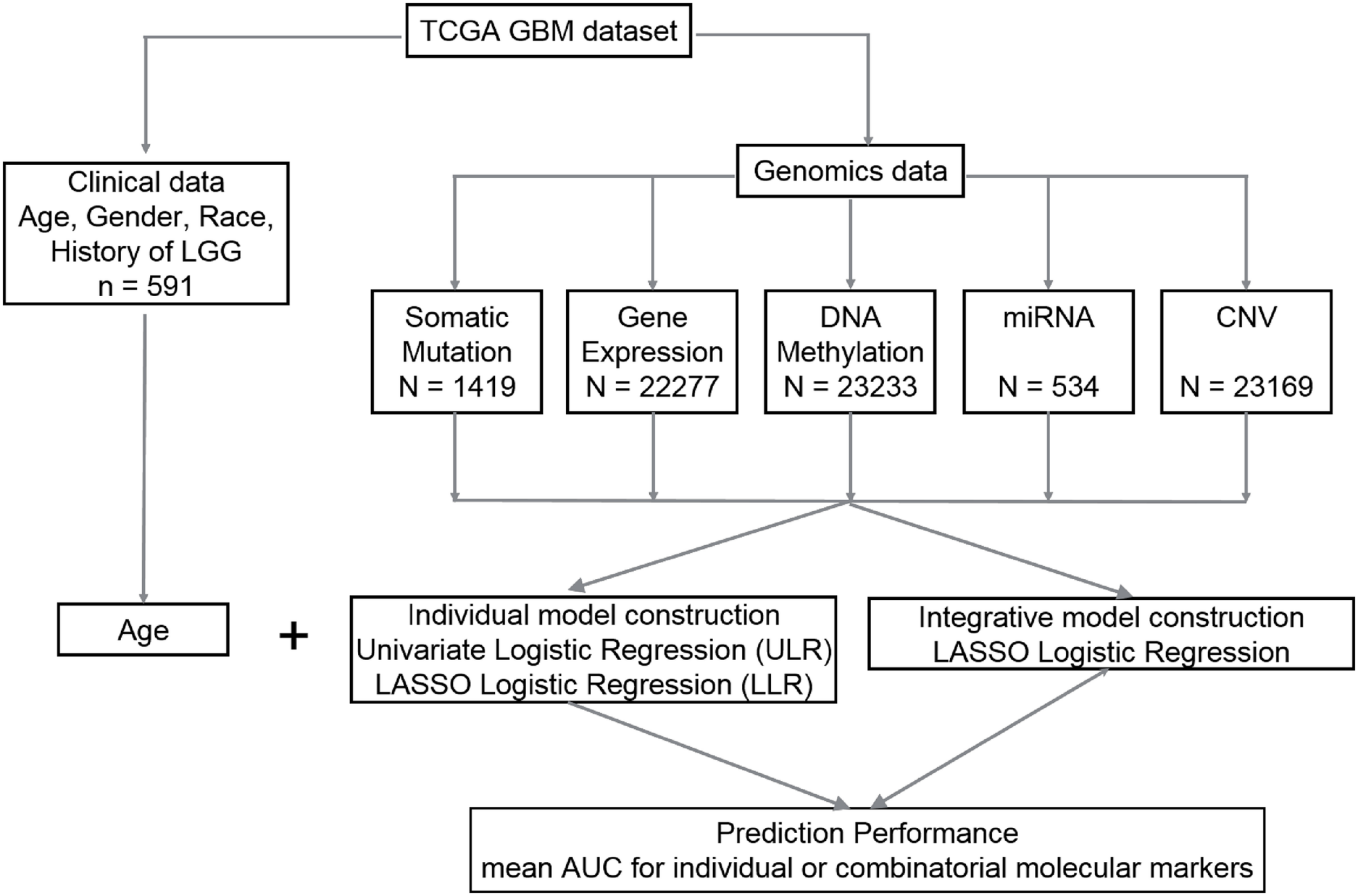
Flow chart with a schematic of the data analysis pipeline used in this study.

Prediction of LTS is a statistical binary classification problem. Models were individually constructed for each molecular data type using both False Discovery Rate (FDR)—adjusted univariate logistic regression (ULR) and Lasso logistic regression (LLR). Integrative models that combine clinical variables with one or more types of molecular profiles were constructed using LLR. The individual and integrative model construction procedures are schematically represented in Fig 1.

1. Univariate logistic regression (ULR). A univariate logistic regression model was fitted for each gene or probe in every class of genomic data, with the genotype at each variant site as a predictor Y = 0,1 (non-LTS vs. LTS) with *p*-values adjusted using a Bonferroni correction for each class of data (in cases with limited data or where no significant associations were found following Bonferonni correction, the less stringent Benjamini-Hochberg adjustment was applied). Significantly LTS-associated features were selected with Bonferroni-adjusted *q* < 0.05. On tests performed on individual traits, the unadjusted *p* < 0.05 was used for feature selection.
2. Lasso logistic regression (LLR). Least absolute shrinkage and selection operator (Lasso) is a penalized multivariable regression model whereby parameter shrinkage and feature selection are done simultaneously [32]. Lasso imposes a penalty on the regression coefficients *β* = (*β*_1_, …, *β*_p_) by restricting the sum of the absolute values (L1 norm) of the coefficients *β*_j_ to values no greater than the shrinkage parameter *λ*. By selecting an appropriate *λ*, a Lasso model can be tuned to include any number of variables in the final regression model; smaller values of *λ* will set more coefficients *β*_j_ to zero, effectively removing them from the model. We implemented Lasso logistic regression using the ‘glmnet’ library in R with the binomial distribution option, reflecting the binary response variable Y. *λ* was selected using 10-fold cross validation so that the model minimizes cross-validation error. Prediction performance was evaluated with Area Under the Receiver Operating Characteristic Curve (AUC) estimation, a commonly used evaluation metric for binary classification. A perfect model will score an AUC of 1, while at the other extreme an AUC near 0.5 reflects models with no predictive power that essentially select *Y* = 0,1 by a random guess.
3. Integrative Models. Integrative LASSO logistic regression models were constructed by using 4 classes of molecular data in combination as predictors of LTS, i.e. clinical information, gene expression levels, DNA methylation scores, CNV counts, and miRNA expression levels were used in combination to identify subsets of molecular markers that were jointly predictive of LTS. To prevent overfitting, initial feature selection was performed for each class of data by selecting all variables with unadjusted *p* < 0.05 in the ULR models; only those genes/probes above the threshold were pooled for multiple regression analysis. We identified a core set of *n* = 212 samples (including 23 LTSs) with all molecular data types represented except for point mutations. Both somatic and germline point mutations were excluded from the combined data sets because no point mutations were significant predictors of LTS in adjusted ULR models and their inclusion would have produced a much smaller sample set since so few tumors contained any particular somatic mutation. Consequently, separate regression analyses were performed with clinical information and point mutation genotypes as predictors of LTS.

A total of 69213 features representing the 4 classes of molecular data from 212 patients were jointly modeled using LASSO logistic regression. Prior to model fitting, each variable was z-transformed to zero mean and unit variance so that variables across different classes of data would be on the same scale. We also constructed regression models using specific combinations of data classes and excluding others (e.g. gene expression + methylation data used to predict LTS while excluding CNV and miRNA data etc), as this allows us to compare predictive performance of models and to determine the marginal effects of incorporating additional data classes on LTS prediction. To evaluate the prediction performance for individual models, we performed tenfold cross-validation and computed the mean AUC over 100 iterations. In every iteration, the data set was divided into 10 subsets, and the LLR was repeated 10 times. One of the 10 subsets was used as the test set and the other 9 subsets were pooled to form a training set in order to compute the average AUC across all 100 iterations. The advantage of this method is that it minimizes the bias from the division of data into training and test sets.

### Imbalanced Sampling and Bootstrapping

Because LTS account for <10% of GBM samples, logistic and LASSO regression analyses of the entire data set by necessity use imbalanced data, which can potentially bias estimation and prediction in logistic regression and machine-learning models [33]. To determine the extent of artifacts introduced by imbalanced data, we performed a bootstrap analysis by downsampling with replacement the non-LTS set to equal the number of LTS samples over 100 replicates (random sampling with replacement of 90% of LTS, 10% of non-LTS). The sensitivity of regression models to downsampling is determined by computing the distributions of AUC values for LLR and logistic regression coefficients for ULR, and compared to the values obtained for models computed from the imbalanced complete data.

### Principal Components Analysis and Hierarchical Clustering

To explore the relationship between LTS GBM and LGG tumors, we represent each sample in a coordinate space defined by the principal components of gene expression and methylation measures. Gene expression data from the AgilentGA4502A microarrays for both GBM and LGG samples was analyzed following Loess normalization and quantile normalization to correct for within and between-array bias, respectively. Significantly differentially expressed genes (DEGs) were identified as follows: a Student’s t-test with Benjamini-Hochberg FDR correction of the *p*-values was performed for each probe to compare mean expression levels between the sample sets. DEGs were identified as those in which the FDR adjusted *q <* 0.01 and the median log fold-change across probes was at least two fold (|log_2_FC| ≥ 2).

Principal Component Analysis (PCA) was performed on four sets of genes: 1. DEGs between LGG and GBM tumors. 2. DEGs between GBM and normal brain tissues. 3. genes whose expression levels were significant predictors of LTS in the ULR models. 4. genes selected for inclusion the LLR model. For each set of genes, the expression values were projected onto principal components 1 and 2, representing each sample’s coordinates in this PC space. Moran's *I*, a measure of autocorrelation, was used to measure the extent to which samples from a defined subset (e.g. LTS patients) cluster together due to similar expression values [34]. This measure was applied to determine the similarity of gene expression or methylation profiles among LTS samples in the coordinate spaces defined by the first two principal components. In the context of this study, a Moran’s *I* value near 0 indicates that LTS expression levels are randomly dispersed among the GBM samples, a value near 1 indicates that the LTS samples are closest to one another in PC space, while a value of -1 indicates a perfectly uniform spacing of LTS and non-LTS samples in PC space (i.e. negative autocorrelation, or a tendency of LTS and non-LTS samples to “alternate” in gene expression space). The same approach was used to study DNA methylation patterns between GBM and LGG with three different gene sets: 1. All genes with methylation probes; 2. ULR selected genes; 3. LLR selected genes.

Additionally, we used unsupervised hierarchical clustering of gene expression values to compare LTS with non-LTS GBM and LGG expression profiles. In this cluster analysis, each sample is represented as a vector of expression values and classified by pairwise Pearson Correlation Coefficient distance.

## Results

### Patient cohort

**Fig 2:**
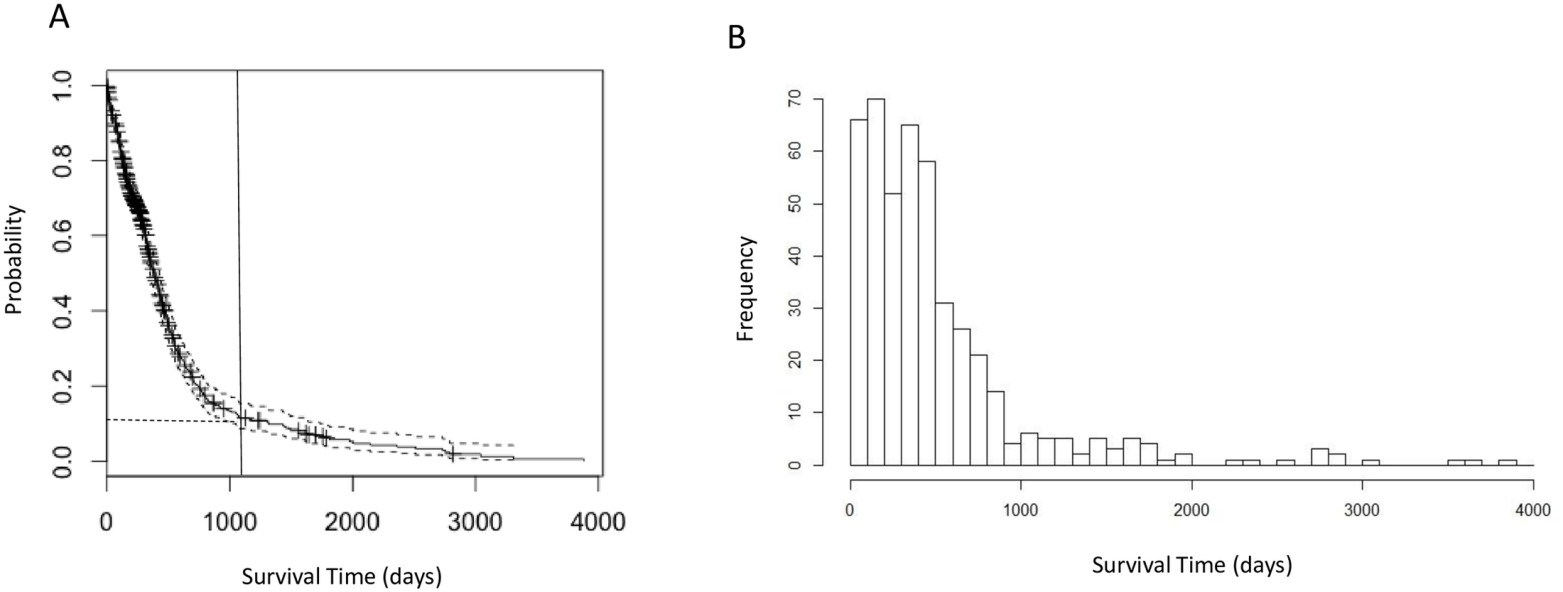
Survival time analyses of GBM patients. a. Kaplan-Meier plot of overall survival analysis of 591 GBM patients. The vertical line indicates the 3-year (1095 days) cutoff for LTS used in following analyses. The horizontal dashed line indicates 7.6% LTS patients corresponding to the cutoff. b. Histogram of survival time (in days) showing that the distribution of survival time is unimodal.

For the 591 patients in this study, the median age at initial diagnosis is 59 years; the patient cohort is 61.4% male (Table 1). We constructed a Kaplan-Meier survival curve (Fig 2A) and estimated the median survival time following initial resection to be 13.9 months (95% CI: 12.9–14.9 months). The survival curve resembles an exponential distribution, indicating a high probability of death within a short period after diagnosis and a comparatively low frequency of LTS. The distribution of survival times is essentially unimodal (Fig 2B), suggesting a homogeneous distribution, rather than a mixture of two or more distributions for LTS and the non-LTS samples. This observation was confirmed by applying Hartigan’s dip test [35](*p* = 0.9877), a distribution-free test for bimodality, which suggests that survival times follow a homogeneous, unimodal distribution.

Among the 591 patients with known vital status and survival times, 44 (7.4%) survived longer than three years—a commonly-used survival milestone for GBM patients [5, 7, 9, 36]–and were classified as “long-term survival” (LTS) patients. Patients with shorter-than-three-year survival time were classified as non-LTS (411, 69.5%). There are a total of 29 patients surviving past the more stringent 4.5 years definition of LTS.

### Regression Analyses of Clinical and Molecular Features

In a logistic regression model that included only clinical and demographical variables, younger age at initial diagnosis, Karnofsky performance score (KPS) and presence/absence of chemotherapy were the three significant predictive factors of LTS (Table 3). While post-operative KPS scores are stronger predictors of patient outcomes than pre-operative scores [37], the TCGA data contain only 76 samples with post-operative/post-adjuvant KPS values, so that pre- and post-operative scores were pooled. In the initial LLR analyses, patient age, KPS, and chemotherapy were considered jointly with the molecular biomarkers as independent variables in multiple regression analyses. Combining KPS and chemotherapy reduces the effective sizes of gene expression and DNA methylation datasets to 277 and 194 samples (due to 28% and 14% missing data in KPS and chemotherapy, respectively), while there was no missing age data.

**Table 3:** Partial regression coefficients for logistic regression model for LTS against clinical and demographical information.

	Estimate	Std error	z value	Pr(>|z|)
Intercept	-2.300	1.9698	-1.168	0.24299
Age	-0.054	0.0135	-4.008	6.12E-05*
Gender (male)	-0.025	0.4015	-0.061	0.95128
LGG history	0.075	1.2110	0.062	0.95034
Race (black)	-0.185	1.1205	-0.165	0.86874
Race (white)	-1.159	0.8813	-1.316	0.18834
Karnofsky score	0.047	0.0170	2.790	0.00526*
Radiation	2.011	1.2393	1.622	0.10473
Chemotherapy	-1.555	0.7191	-2.163	0.03054*

Age at initial diagnosis, gender, ethnicity, presence/absence of prior LGG history and presence/absence of chemo and radiotherapy are used as independent variables in the model. * Independent covariates with statistically significant partial regression coefficients are indicated with ‘*’ (*p* < 0.05).

“Estimate” is the coefficient associated with the variable; “Std.Error” is the standard error associated with these estimates; “Pr (>|z|)” is the *p*-value associated with the z-value.

We evaluated the regression models with or without the inclusion of KPS and chemotherapy, and found that they converged to a set of predictors that included age but not KPS or chemotherapy (S1 Table), presumably reflecting the strong (*r* = -0.323, *p* = 1.93E-9) correlation between KPS and age, as well as the small fraction of patients (13.5%) who did not receive chemotherapy. Moreover, an identical set of predictor biomarkers and nearly identical coefficients were obtained from LLR that initially included KPS and chemotherapy as from models where only age is initially included on the same set of samples. Because of this and the reduction of sample size, age is the only clinical variable included in subsequent regression analyses.

ULR identified 10 somatic mutations as predictors of LTS with p < 0.05 (none of which are statistically significant after Bonferroni or Benjamini-Hochberg adjustment of p-values), among these are mutations in genes whose somatic variants are well-known to correlate with GBM survival time such as *IDH1* and *PRSS1* (S2 Table). LLR identified 13 somatic mutations as significant (Table 4 and S3 Table). Most of the significant LLR mutations are located in different genes from those identified from ULR, except for mutations in IDH1 and the mRNA splicing gene DHX16. We note that IDH1 somatic mutations are the most significant predictors of LTS in both cases, with unadjusted p = 3.2E-3 in ULR and β = 1.10 in LLR. This is consistent with the occurrence of non-synonymous mutations in IDH1 in 16.67% of the LTS patients versus 1.19% of the non-LTS patients in the TCGA sample set, corresponding to an odds ratio of 16.03 (p = 6.8E-3). There are 39592 germline mutations (SNPs) with unadjusted p < 0.05, although none are significant under either Bonferroni or Benjamini-Hochberg adjustments, even when the adjustment is restricted to the set of mutations in the exome. In LLR analysis, we identified 8 SNP genotypes with nonzero regression coefficients. The strongest associations are for mutations in the *B3GALT5* (a Beta-galactosyltransferase gene) gene and the TGS1 (trimethyguanosine synthase), with β = -0.28, -0.18, respectively, indicating that the wild type genotypes at these loci are weakly predictive of LTS (AUC = 0.52, 95% CI: 0.44–0.60, Table 5).

**Table 4:** List of significant predictor genes in LLR using single classes of molecular data.

	Genes (Positive Association)	Genes (Negative Association)
Germline Mutation	*B3GALT5*, *TGS1*	None
Somatic Mutation	*IDH1*	None
Gene Expression	*MLNR*, *PI15*, *NOS3*, *NEUROG1*, *MFI2*	*MST1L*,*CRLF2*
DNA Methylation	*HS1BP3*, *CDKN1B*, *TMED10*, *PURB*, *RSPO3*, *LETMD1*, *STX17*	*TNS4*, *C6orf48*, *SNORD48*, *LLGL1*, *VIM*, *NLRP4*, *CXorf23*
Copy Number Variation	AY289773,HPR	*AL713660*, *DUSP28*
miRNA	None	*hsa-miR-222*

Only predictor genes with relative large beta are shown here (i.e. |β| > 1 for somatic mutation, gene expression, DNA methylation, CNV and miRNA; |β| > 0.1 for germline mutation). For a complete list, see S3 Table.

**Table 5:** Prediction performance of individual molecular type under LLR, as measured by AUC.

	Unbalanced		Balanced	
Type of Variable	mean AUC	Std	mean AUC	Std
Age	0.8034	0.0150	0.8070	0.0901
Germline Mutation	0.5169	0.0395	0.5490	0.0832
Somatic Mutation	0.6156	0.0354	0.6451	0.0724
Gene Expression	0.7385	0.0322	0.6665	0.0706
DNA methylation	0.8387	0.0341	0.6747	0.0962
miRNA	0.7350	0.0272	0.6577	0.0724
CNV	0.7002	0.0232	0.6785	0.0599

The last two columns are the mean and standard deviations of AUC under 100 bootstrap permutations (i.e. downsampling the ~10% of non-LTS cases and 90% of LTS so that the number of samples is equal to that in LTS).

For the gene expression data, there are 38 significant LTS predictors with FDR-adjusted ULR (S2 Table) vs 94 with LLR (Table 4 and S3 Table). Functional enrichment analysis of the 478 ULR significant predictor genes (*q* < 0.05) found a significant enrichment in phosphoproteins (1.32 fold enrichment, *p* = 1.6E-04) and genes in acetylation pathways (1.78 fold enrichment, *p* = 4.39E-06) (S4 Table). In contrast, the 94 significant predictor genes in LLR analysis did not identify enrichment with respect to any known KEGG pathway or structural/functional classes of genes. Among the genes whose expression levels are positively associated with LTS are *NOS3* (nitrous oxide synthase, a known regulator of blood pressure and other vascular function) [38] and the neurogenin *NEUROG1*, a transcription factor regulating growth of neurons [39], indicating that the upregulation of these genes is linked to a higher probability of LTS.

Among the 4 classes of genomic data, DNA methylation is the strongest predictor of LTS with the highest mean AUC (AUC = 0.84, CI: 0.78–0.90) in LLR models (Table 5), which was confirmed through 100 replicates of 10-fold cross validation. Indeed, methylation is an even stronger predictor of LTS than age (i.e. AUC = 0.80, CI: 0.77–0.830). This is the case even though there are fewer samples in the methylation data set than for gene expression, miRNA, and CNVs. We found 38 methylation probes that are significant predictors of LTS in adjusted ULR models (S2 Table) vs. 43 in the LLR model (Table 4 and S3 Table). Genes with Lasso regression coefficients |β| > 10 include *LETMD1*, a known oncogene [35], the known tumor suppressor *CDKN1B* [36], as well as several other genes whose variants have been linked with other cancers, such as *RSPO3* [37]. All of these genes are positively associated with LTS, indicating that their hypermethylation is predictive of improved patient outcomes. *TNS4*, whose oncogenic role has been documented for colorectal and other cancers[38], has the strongest negative association with LTS, suggesting that hypomethylation of this gene is predicts LTS (see Table 4 and S3 Table for a summary of genes that significantly predict LTS in LLR models). We remark that there is no significant association of *MGMT* promoter region methylation with LTS in LLR models, nor is methylation of this region a significant LTS predictor in a ULR model following FDR correction. However, the association between *MGMT* hypermethylation and LTS is significant in a ULR model (*p* = 0.036) without adjustment. There was no overlap between the set of genes that were differentially expressed between LTS and non-LTS and those that were differentially methylated.

A single microRNA was found to be significantly predictive of LTS with either the ULR or LLR analyses (Table 4, S2 and S3 Tables), namely *hsa-miR-222*. The regression coefficient of *hsa-miR-222* expression levels on LTS is -0.169, indicating that downregulation of this miRNA is associated with LTS. There are 11 and 29 significant CNV probes prediction LTS with ULR and LLR, respectively (Table 4, S2 and S3 Tables). The strongest association of CNVs with LTS (|β| > 1) in the LLR data included the oncogene *DUSP28* [40] (a negative association, indicating that deletion in this gene is predictive of LTS), as well as a positive association with *HPR* CNVs (i.e. duplication at this locus is correlated with LTS). Mutations in *HPR* have been documented in the literature as predictors of recurrent breast cancer [41]. *STAM*, encoding a signal transduction adapter molecule, was found to be a significant predictor in both gene expression and CNV analyses. Higher expression and amplification of this gene was associated with LTS (S3 Table), suggesting that the genomic amplification of *STAM* might lead to the upregulation of gene expression.

### Imbalanced Sampling and Bootstrapping

The creation of balanced LTS vs. non-LTS data sets by downsampling did not substantially change the AUCs of the regression models. As can be seen in the last 2 columns in Table 5, the bootstrapped mean values of AUC are nearly identical for some data types (e.g. CNV and ULR using patient age), slightly higher for some data sets (e.g. somatic mutation and germline mutation) and somewhat lower for others (e.g. expression levels and methylation). These results indicate that the fit of models to data is not an artifact of imbalanced sampling.

### Integrative Model Construction

In terms of AUC, combining one or more classes of genomic data with age in an LLR does not strongly enhance prediction of LTS when compared to age alone. It can be seen in Table 6 that combining age with methylation and microRNA expression data only marginally improves AUC, while AUC actually decreases when CNV counts or gene expression are combined with age, which is consistent with the relatively lower prediction performance of gene expression and CNV for individual models. The strongest improvement in prediction occurs when age is combined with the single significant miRNA *hsa-miR-222*, i.e. (AUC = 0.87, 95% CI: 0.83–0.91). The same is true when multiple classes of genomic data are combined in a single regression model, e.g. expression+methylation+microRNA data combined with age give virtually identical AUC values to age alone, indicating that pooled biomarker data does not outperform individual classes of biomarkers in LTS prediction, perhaps as a consequence of increased number of features. Applying a more stringent cutoff (4.5 years) for LTS classification does not change the results qualitatively in terms of either the significant predictors or the magnitude of AUC (prediction of miRNA expression and CNV is moderately enhanced, while diminishing for gene expression data, presumably due to fewer LTSs). This indicates that the results of the regression analyses are not strongly predicated on the choice of cutoff time used to define LTS.

**Table 6:** Prediction performance (area under curve, AUC) of integrative models.

Combinations	cutoff = 3 y		cutoff = 4.5 y	
	mean AUC	Std	mean AUC	Std
age	0.8033	0.0170	0.8023	0.0150
age+exp	0.7246	0.0404	0.6350	0.0630
age+met	0.8067	0.0350	0.8095	0.0467
age+mir	0.8711	0.0202	0.9028	0.0262
age+cnv	0.7164	0.0547	0.7638	0.0638
age+exp+met	0.8095	0.0343	0.7980	0.0451
age+exp+mir	0.7470	0.0429	0.6761	0.0591
age+exp+cnv	0.6918	0.0475	0.6554	0.0596
age+met+mir	0.8126	0.0411	0.8012	0.0421
age+met+cnv	0.8034	0.0416	0.8128	0.0422
age+mir+cnv	0.7777	0.0481	0.8193	0.0619
age+exp+met+mir+cnv	0.8107	0.0379	0.8022	0.0408

These models combine age with one or more molecular types with LTS cutoff at 3 year (i.e. 7.6%) or 4.5 year (5%).

The principal value of constructing integrative LLR models lies in the fact they identify molecular markers that are jointly significant predictors of LTS which are not individually predictive in ULR, nor predictive in LLR when applied to a single class of data (see Table 7). For example, in LLR analysis of methylation probes alone, methylation of the oncogene *BRAF* does not appear as a significant predictor, whereas in the integrative model it has β = 4.62. While *CAV1* (caveolin 1, a plasma membrane protein and tumor suppressor gene) appears in the LLR model, its β = 11.34 value is much larger in the joint regression model than in LLR on methylation data alone. Downregulation or loss of function in *CAV1* has previously been documented as a determinant of aggressiveness in GBM tumor growth [42].

**Table 7:** List of significant predictor genes in integrative LLR models, with various combinations of data classes.

Probe	Gene Symbol	Beta	In individual model
205742_at	*TNNI3*	0.0987	NO
205923_at	*RELN*	0.0024	NO
215443_at	*TSHR*	0.0286	YES
216512_s_at	*DCT*	0.2786	NO
cg05790999	*HS1BP3*	34.506	YES
cg07964538	*CAV1*	11.638	YES
cg09307279	*GLT8D1;SPCS1*	12.851	NO
cg10141022	*BRAF*	4.6218	NO
cg12927772	*C9orf64*	2.8326	YES
cg12989642	*PURB*	12.553	YES
cg18672421	*TMED10*	3.041	YES
cg19133903	*AVPI1*	0.553	NO
cg21982518	*TMC7*	1.3812	NO
cg25913233	*SPARC*	2.1799	NO
cg26864028	*EPOR*	0.086	YES

All but one of the gene expression probes that appear in the integrative LLR model are not significant LTS predictors for LLR on expression data alone. We remark, however, that most of these expression probes are only weakly predictive of LTS in the joint model, with β≤0.28 (the strongest association is with expression levels of *DCT*, dopachrome tautomerase). This result is consistent with methylation being the strongest individual predictor of LTS in both integrative models and in models that only incorporate a single class of data.

### Relationship of LTS to LGG

**Fig 3:**
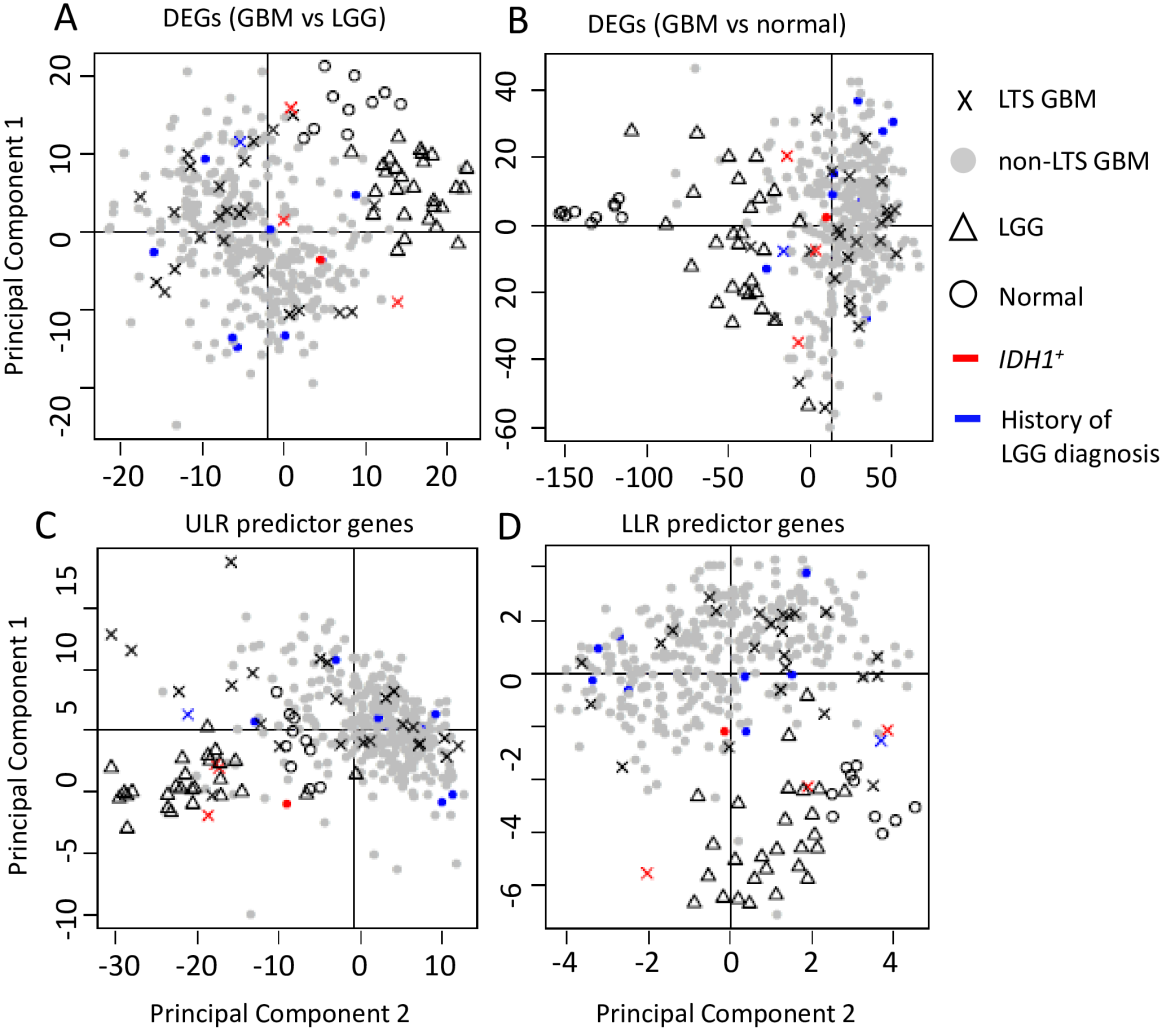
Scatterplot of the first two principal components of gene expression data. PCA analyses were performed on a. DEGs between GBM and LGG (N = 491 genes). b. DEGs between GBM and normal controls (N = 4801). c. ULR predictor genes (N = 94). d. LLR predictor genes (N = 38).

**Fig 4:**
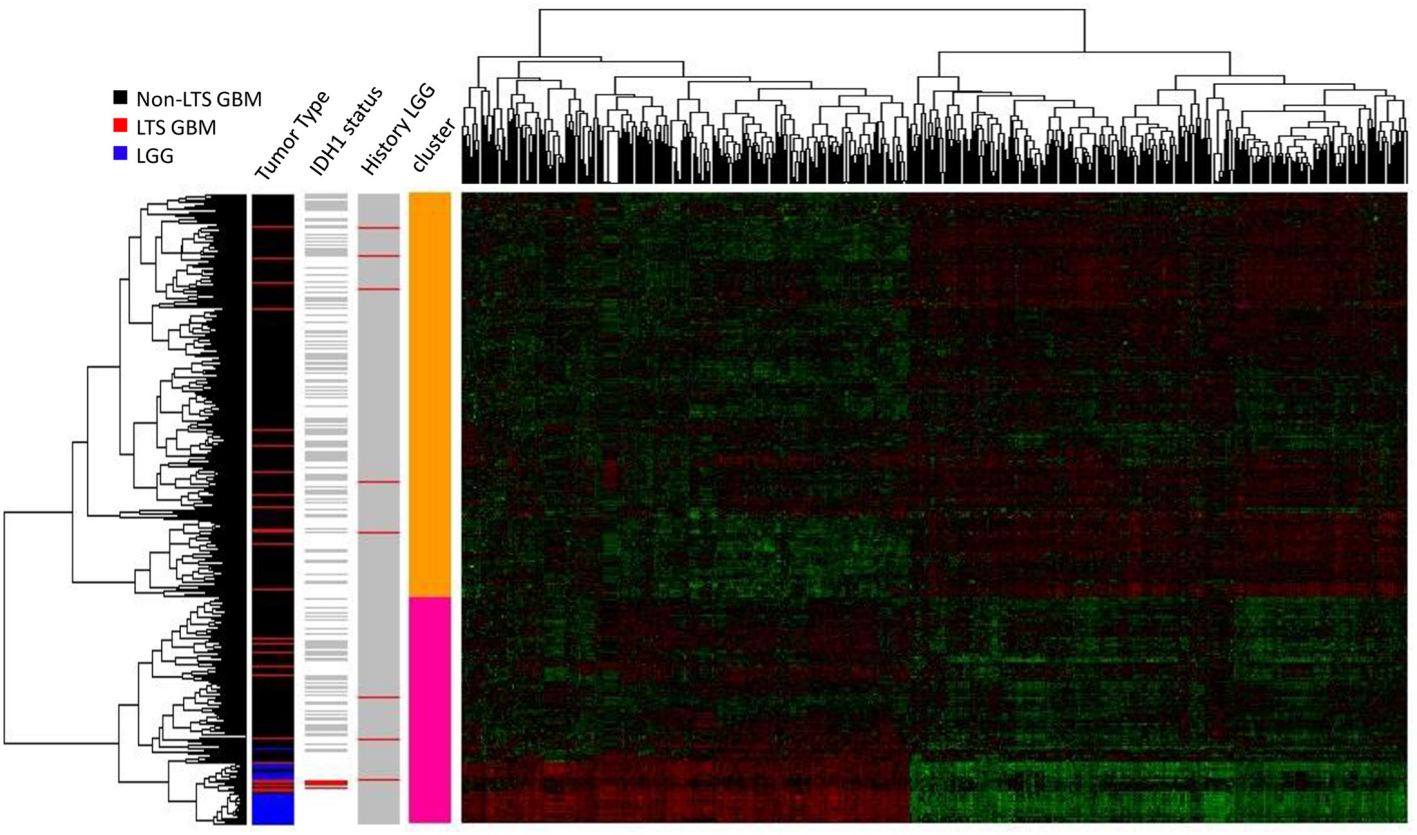
Heatmap of the gene expression levels in LTS GBM, non-LTS GBM and LGG samples (N = 383 genes). Hierarchical clustering (HC) on the expression levels of DEGs between GBM and LGG (N = 491) was used to classify the samples, with a row dendrogram (clustering of samples) based on Pearson correlation coefficient, the column dendrogram on a Spearman correlation coefficient. In the row-side color bars of *IDH* status and LGG history, red indicates *IDH1*^+^ or history of LGG diagnosis; grey indicates *IDH*1^-^ and no history of LGG diagnosis, respectively; white indicates that no data is available for the sample.

The PCA analyses reveal that the GBM, LGG, and normal samples have distinct profiles among their DEGs (Fig 3A and 3B). LGG and (all) GBM samples are distinct clusters in the PCA scatterplots (Moran’s *I*~0.1, *p* << 0.01 for LGG vs. GBM), and all LGG tumors share common nodes in the hierarchical clustering analysis discussed below (Fig 4). In contrast, LTS samples are interspersed among the non-LTS GBM samples (Moran’s *I* near 0, *p* > 0.1 in Table 8); methylation scores (Table 9) produces qualitatively similar results. Together, these results suggest that there is no “hallmark” gene expression profile for LTS GBM, consistent with the lack of association between expression profile subtype and LTS described in [21]. These observations all indicate weak mutual similarity between expression profiles of LTS GBMs, i.e. the majority of LTS GBMs have gene expression patterns that more closely resemble non-LTS GBM than they do the profiles of other LTS patients. Not surprisingly, if we consider the gene sets that are significant predictors of LTS in ULR and LLR models, we do find separation and autocorrelation among LTS samples, the Moran’s *I* values have *p* effectively 0 for genes identified ULR and LLR, respectively. This could also be seen by the greater “clumping” of LTS samples in PCA space and in the dendrograms (S1 and S2 Figs).

**Table 8:** Moran's I for each binary category (e.g. LTS vs. non-LTS).

DEGs (LGG vs GBM)	Observed	Expected	Std	*p* value
LGG vs GBM	0.2991	-0.0029	0.0055	0
LTS vs nLTS	0.0051	-0.0029	0.0053	0.1340
normal vs tumor	0.1989	-0.0026	0.0049	0
DEGs (GBM vs normal)				
LGG vs GBM	0.1238	-0.0027	0.0052	0
LTS vs nLTS	-0.0004	-0.0029	0.0056	0.6525
normal vs tumor	0.2980	-0.0026	0.0053	0
ULR genes				
LGG vs GBM	0.2863	-0.0029	0.0066	0
LTS vs nLTS	0.0241	-0.0029	0.0060	7.84E-06
normal vs tumor	0.1057	-0.0026	0.0057	0
LLR genes				
LGG vs GBM	0.1047	-0.0029	0.0068	0
LTS vs nLTS	0.0204	-0.0029	0.0068	0.0007
normal vs tumor	0.1345	-0.0026	0.0060	0

The statistics are computed over the coordinates of the first two principal components of gene expression.

**Table 9:** Moran's I for each binary category (e.g. LTS vs. non-LTS).

All genes	Observed	Expected	Std	*p* value
LGG vs GBM	0.3951	-0.0026	0.0060	0
LTS vs nLTS	0.0027	-0.0036	0.0068	0.3513
normal vs tumor	-0.0030	-0.0026	0.0005	0.4193
ULR genes				
LGG vs GBM	0.3373	-0.0026	0.0059	0
LTS vs nLTS	0.0033	-0.0036	0.0071	0.3356
normal vs tumor	-0.0033	-0.0026	0.0005	0.2393
LLR genes				
LGG vs GBM	0.3412	-0.0026	0.0066	0
LTS vs nLTS	0.0014	-0.0036	0.0086	0.5612
normal vs tumor	-0.0033	-0.0026	0.0005	0.2495

The statistics are computed over the coordinates of the first two principal components of DNA methylation.

In the hierarchical clustering analysis, selecting a twofold partition generates one subtree that consists solely of GBMs and another that contains both GBM and all LGG samples (i.e. all LGG samples are defined by a single node in this subtree, as shown in Fig 4). LTS samples occur in both subclusters, with a disproportionate representation of LTS tumors in the subtree containing the LGG. Specifically, 14 of the 28 LTS GBM samples occur in the subtree that also contains LGG, versus 93 of the 318 non-LTS GBM samples (OR = 1.72, *p* = 0). However, most of the neighbors of LTS in the dendrogram are non-LTS GBMs, even for those in the subtree that contains LGG (as in the scatterplot). The same is true of GBMs in patients who have a prior history of LGG, that is, known secondary GBMs occur throughout the dendrogram and show no specific association with LGG expression profiles. In fact, only a single LTS has a documented prior history of LGG (corresponding to an insignificant OR = 1.04 for LTS association with LGG). From both the hierarchical clustering and PCA analyses, we conclude there is no significant association between progression from LGG and subsequent LTS in GBM.

A closer examination of *IDH1* mutation status in showed that all 5 *IDH1*^*+*^ GBMs (including 3 LTSs and 2 non-LTSs) cluster with LGGs when the dendrogram was partitioned into two clusters (Fig 4, row-side color bar on the right), indicating a similarity in gene expression and methylation profiles between *IDH1+* genotypes and LGGs. However, while *IDH1+* is a significant predictor of LTS, the majority of LTS cases in the TCGA data set are *IDH1-* wildtype. Furthermore, none of the *IDH1+* genotypes was in a GBM with a prior LGG history.

Most of the LTS samples are similarly interspersed with non-LTS GBM’s in the PCA scatterplots, only a small subset of LTS samples cluster with LGGs. The lack of a strong overall LGG-like “signal” in LTS samples can be seen most clearly from the comparison of centroids for PCA 1+2 scores over the different gene sets (S5 Table), where the LTS distances to LGG were much greater than to non-LTS GBM. Indeed, except for the small subset of genetic markers that are predictive of LTS, the absence of significant autocorrelation among LTS samples further argues against LTS corresponding to a biologically unique and qualitative distinct class of GBM pathology, and against their proposed molecular affinity with LGG features.

Moreover, GBM samples from patients with prior LGG history do not cluster together in the dendrogram, nor do these known secondary GBMs cluster with LGGs, arguing against the hypothesis of that secondary GBMs retain LGG-like molecular profiles. Previous analyses of gene expression patterns have identified at least four subtypes of GBM [43–45], including secondary (proneural) GBM, as well as the mesenchymal, classical and neural GBM subtypes. However, we found no significant association between LTS and the proneural subtype (based on TCGA classification of samples), the OR = 1.21, *p* = 0.83. This lack of association of LTS with subtype is consistent with the observation that most LTS samples do not share a common node in the dendrogram nor a specific affinity with LGG.

**Fig 5:**
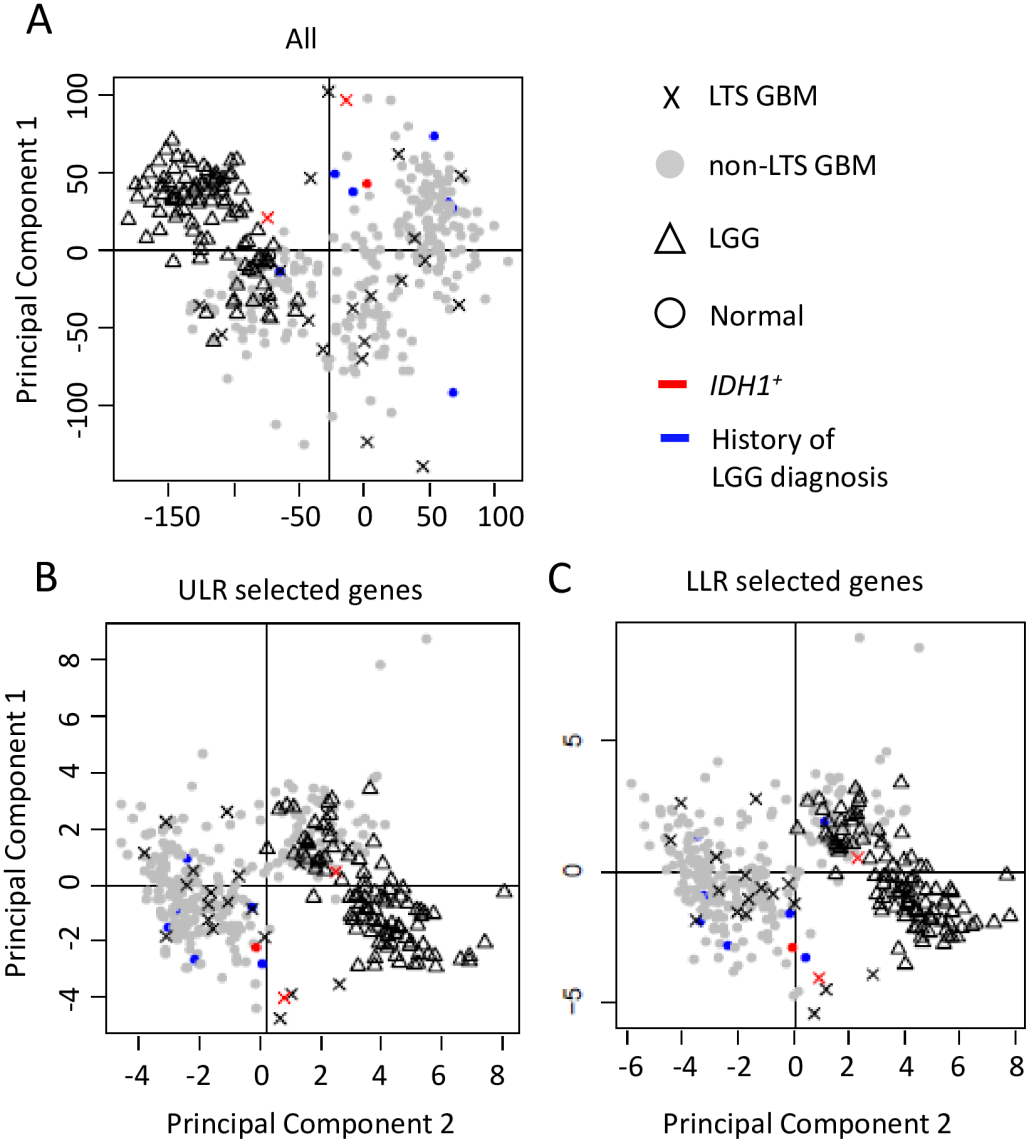
Scatterplot of the first two principal components of the DNA methylation data. PCA analyses were performed on a. All probes (*N* = 23233). b. ULR predictor genes (*N* = 38). c. LLR predictor genes (*N* = 43).

**Fig 6:**
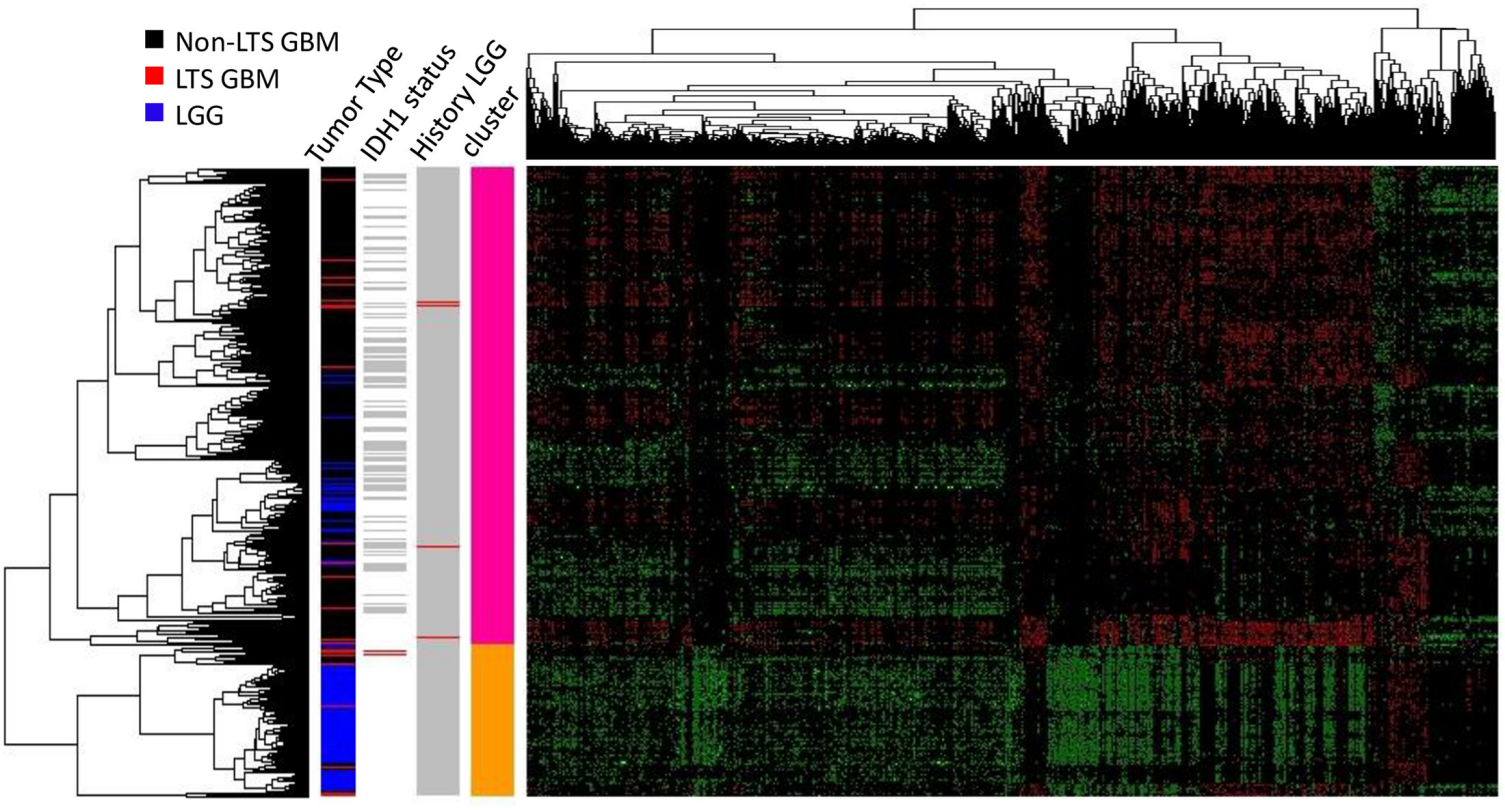
Heatmap of the DNA methylation levels in LTS GBM, non-LTS GBM and LGG samples (N = 383). Hierarchical clustering (HC) on the DNA methylation levels of GBM and LGG (N = 23233) was used to classify the samples. The row dendrogram (clustering among samples) is based on Pearson correlation coefficients and the column dendrogram on Spearman correlation coefficients. In the row-side color bars of *IDH* status and LGG history, red indicates *IDH1*^+^ or history of LGG diagnosis; grey indicates *IDH*1^-^ or no history of LGG diagnosis; white indicates no data available for the sample.

Similar trends are observed with the methylation data (Figs 5 and 6, S6 Table), finding no association between LTS and LGG samples with respect to methylation scores. Furthermore, the tendency of LTS samples to co-occur in a single cluster is even weaker for the methylation profiles. The Moran’s *I* autocorrelation measures are statistically insignificant for LTS methylation scores with respect to non-LTS GBM (Table 9), even when the LLR subset of genes are considered separately. These results were unexpected in view of the fact that methylation is a stronger predictor of LTS in LLR models than expression profiles, which is probably a consequence a relatively small subset of the LTS samples with very similar expression profiles (high autocorrelation) in the LLR-selected genes.

## Discussion

The principal goal of this study was to determine whether LTS GBM tumors have genomic features that distinguish them from those found in patients with more typical survival times, i.e. to evaluate whether they constitute a biologically distinct subclass of high-grade gliomas with unique molecular characteristics, and to evaluate the relationship between LTS GBMs and LGGs.

In spite of the limitations on statistical power due to comparatively small samples of LTS patients and incomplete clinical data, our analyses identified molecular biomarkers that significantly predict LTS, several of which have been documented in the literature as predictors of improved response to chemotherapy and overall improved patient prognosis. For example, among the somatic point mutations, non-synonymous mutations in *IDH1* were the strongest predictors of LTS in ULR and LLR models (Table 4), consistent with the significantly higher proportion of *IDH1* mutated patients observed in the LTS vs. non-LTS data sets. However, even the association of LTS with *IDH1* mutations is weak, i.e. even though most of *IDH1+* genotypes are LTS, most LTS cases are *IDH1*-. The low predictive performance of both somatic and germline mutations generally is in agreement with emerging clinical data suggesting that *IDH1* is a only weak predictor of LTS in GBM, as survival beyond the fourth year can occur in patients without *IDH1* mutations [12, 21]. Similarly, miR-222, the only differentially expressed miRNA identified as a predictor of LTS, has been previously documented in the literature [46] as predictor of GBM prognosis due to its role as a regulator of the *PUMA* [47], a P53 mediated regulator of apoptosis. Higher levels of *PUMA* protein are associated with increased apoptosis and, consequently, lower growth rates in GBM tumors. Upregulation of *miR-222* is linked to repression of *PUMA* and higher tumor proliferation, consistent with LTS being negatively associated with *miR-222* expression levels.

The scarcity of somatic mutation genotypes as predictors of LTS is largely the result of most somatic mutations occurring in few (2–3) samples in the data set. On the other hand, given the abundance of high frequency variant germline genotypes, the small number of germline mutation predictive of LTS is somewhat surprising, since genome wide association studies (GWAS) [48, 49] have identified inherited SNPs that predispose individuals to GBM. None of these GWAS-identified SNPs appeared as a significant predictor in our regression models. These results suggest that there are few if any inherited (familial) mutations that predict LTS in GBM patients, or that their effects are comparatively weak against the much stronger signal of variation among GBM types and the contribution of clinical variables such as age to patient survival.

Although mutational genotypic markers for LTS are limited, we did identify gene expression phenotypes, epigenetic markers, and copy number variant genotypes that are significantly predictive of LTS, with the exception of DNA methylation. The fact that DNA methylation has comparable or better predictive power than age is probably related to the coordinated regulation of aging and DNA methylation patterns [50]; the same is true for regression models of LTS combining age with methylation. While previous analyses have shown that combining clinical predictors with gene expression alone best predicts survival time [25], our results found the weakest prediction when age is combined solely with gene expression data, and strongest when combined with miRNAs (in spite of the limited number of miRNAs). This discrepancy is potentially due to different choices of algorithms (i.e. feature selection approach, shrinkage parameter optimization) and/or the nature of the model (i.e. response variable: hazard proportion ratio vs. binary response, as well as our use of integrative regression models) [51].

Apart from this comparatively small set of genomic markers, there is no strong genomic “signature” that unambiguously distinguishes LTSs from other GBM. No molecular markers unique to LTS were identified, and there wasn’t the large-scale statistical difference in either gene expression or methylation patterns such as those observed between GBM and LGG. This result is consistent with the observed unimodality of the survival time distribution for the GBM patients (Fig 2A and 2B). If LTS patients represented a biologically distinct subclass of cases, one would expect the distribution of survival times to resemble a bi or multimodal mixture, when in fact the distribution of time until death is basically monotonically decreasing for survival times greater than the mode.

Furthermore, while there are biomarkers that significantly predict LTS in logistic regression models, there are no molecular profiles that strongly define LTS as a distinct subclass in the way that LGGs are molecularly distinct from GBM. This is evident from the occurrence of LTS samples throughout PCA scatterplots, and the lack of a single node or subtree subtending most or all LTSs in dendrograms. Such results suggest that there are many patterns of gene expression and methylation that lead to LTS phenotypes. Similarly, the lack of a consistent molecular signature among secondary GBMs, or a shared signature between secondary GBM and LTS implies that secondary GBMs are not associated with improved patient outcomes in comparison to primary GBM.

Finally, this study found no tendency among LTS GBM cases to resemble the molecular profiles of LGG, which argues strongly against LTS cases being misdiagnosed LGGs. This observation, together with a lack of an association between LTS and secondary GBM, also suggests that LTS in GBM is not a consequence of the retention of LGG-like biological features in high-grade glioma tumor cells. Our finding that only *IDH1+* GBMs have expression profiles resembling LGG may indicate that *IDH1* mutated GBMs are either misidentified LGGs or represent a unique, LGG-like pathology among high-grade gliomas, this observation does not account the majority of LTS cases. The fact that the overall gene expression and methylation profiles of LTS tumors lack a unique molecular signature and don’t show a significant similarity to LGGs simply indicates that there are multiple genomic paths to similar clinical phenotypes and patient outcomes, just as there are multiple genetic and epigenetic paths to cancer. We anticipate that the molecular correlates of different categories of LTS will be further elucidated once larger data sets become available.

## Supporting Information

S1 Fig Heatmap of the gene expression levels for LTS GBM, non-LTS GBM and LGG samples (N = 383). Hierarchical clustering (HC) on the expression levels of ULR predictor genes (N = 38) was used to classify the samples. The row dendrogram, showing the relationship among samples, was based on Pearson correlation coefficients, thecolumn dendrogram on Spearman correlation coefficients. In the row-side color bars of *IDH* status and LGG history, red indicates *IDH1*^+^ or history of LGG diagnosis; grey indicates *IDH*1^-^ or no history of LGG diagnosis; white indicates no data available for the sample.
(TIF)

S2 Fig Heatmap of the gene expression levels for LTS GBM, non-LTS GBM and LGG samples (N = 383). Hierarchical clustering (HC) on the expression levels of LLR predictor genes (n = 94) was used to classify the samples. The row dendrogram (clustering of samples) is based on Pearson correlation coefficient and column dendrogram on Spearman correlation coefficient. In the row-side color bars of *IDH* status and LGG history, red indicates *IDH1*^+^ and a history of LGG diagnosis, respectively; grey indicates *IDH*1^-^ or no history of LGG diagnosis, respectively; white indicates that no data is available for the sample.
(TIF)

S1 Table Comparison of LLR models with and without the combination of KPS and chemotherapy with gene expression and age, as well as LLR models with DNA methylation and age as independent variables. The genes with non-zero penalized regression coefficients (*β*) in Lasso regression model are listed in the table.
(XLSX)

S2 Table List of features that are statistically significant associations with LTS GBM under ULR model. For somatic point mutation, genes with unadjusted *p* < 0.05 are shown. For the other classes of data, genes with adjusted *q* < 0.05 (Bonferroni correction, i.e. pbonf) were shown. “Estimate” is the coefficient associated with the variable; “Std.Error” is the standard error associated with these estimates; “Pr (>|z|)” is the *p*-value associated with the z-value.
(XLSX)

S3 Table List of significant molecular predictors selected with LLR. Genes with non-zero penalized regression coefficients (β) in Lasso regression model are shown. (XLSX)

S4 Table Functional annotation terms associated with LTS in GBM patients. Annotation clusters with adjusted *p*-value (Benjamini-Hochberg) q < 0.05 for related annotation terms are shown. Fold enrichment is the ratio of annotation terms of LTS predictors to those of all genes.
(XLSX)

S5 Table Centroid distance table for PCs of gene expression. (XLSX)

S6 Table Centroid distance table for PCs of DNA methylation. (XLSX)

## Abbreviations

GBM: Glioblastoma multiforme
LTS: Long-term survival
LGG: Low grade glioma
CNV: Copy number variation
TCGA: the Cancer Genome Atlas
PCA: Principal component analysis
LLR: Lasso logistic regression
ULR: Univariate logistic regression
